# Chemical Footprinting: Identifying Hidden Liabilities in Manufacturing Consumer Products

**DOI:** 10.1289/ehp.123-A130

**Published:** 2015-05-01

**Authors:** Lindsey Konkel

**Affiliations:** Lindsey Konkel is a Worcester, MA–based journalist who reports on science, health, and the environment.

In an unassuming low-rise in the Boston suburbs, Mark Rossi tinkers with a colorful dashboard on his laptop screen while his border collie putters around his feet. Rossi is the founder of BizNGO and Clean Production Action, two nonprofit collaborations of business and environmental groups to promote safer chemicals. He’s also the creator of tools that he hopes will solve a vexing problem—how to get a handle on companies’ overall toxic chemicals usage.

**Figure:**
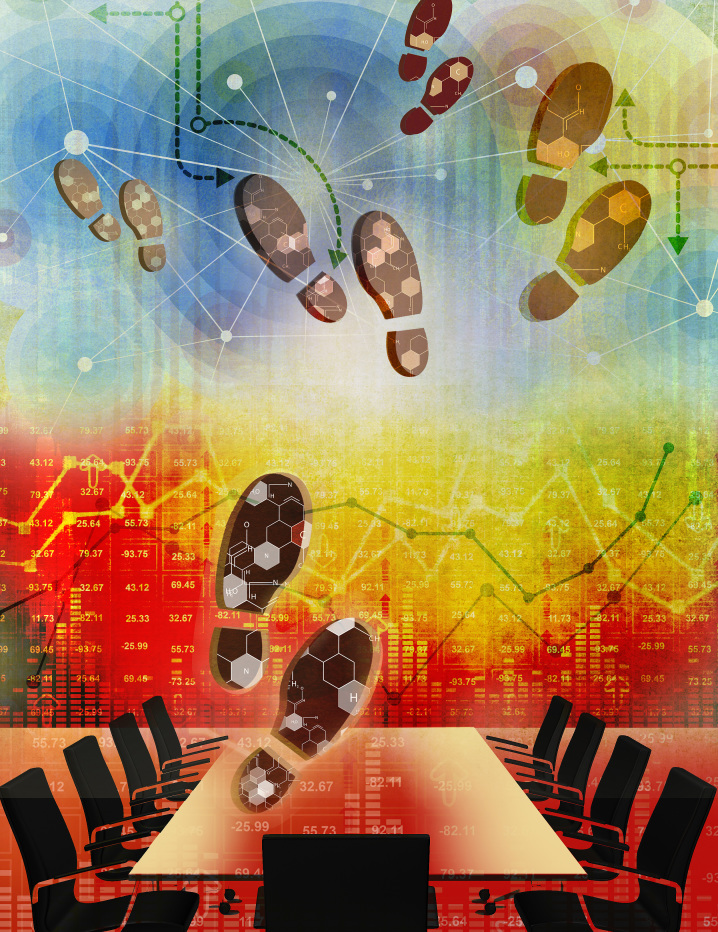
Hidden liabilities arise when companies don’t know or don’t disclose the chemicals in their products and supply chains—omissions that can create uncertainty for investors. A new tool gives companies a way to talk about their performance in toxic chemicals management. © Roy Scott

Consider the screen of Rossi’s laptop. Chances are the company that manufactured the product has crunched the numbers on the total amount of carbon, water, and land associated with getting it into the office—from the manufacturing of the electronic components to the packaging and transportation to retail outlets. But the total amount of toxic chemicals that contributed to the screen’s design and production might be a more difficult question to answer.

“There’s a huge gap in sustainability metrics surrounding chemicals and health. We’re trying to fill  that gap,” Rossi says.

Corporate chemicals management policies have traditionally revolved around compliance with government regulations—making sure certain chemicals don’t exist in products over a mandated threshold. But simply being in compliance may not be enough to protect a company from hidden chemical liabilities in products as regulations shift and consumers, advocates, and investors demand increasing levels of transparency, Rossi explains. New frameworks are now emerging to assess a company’s chemical footprint.

## Birth of a Concept

The concept of “footprinting” developed in the 1990s as a way to capture and quantify the impact of human actions on the environment. The goal was to facilitate public discussion of complicated phenomena by presenting it in a simple, accessible form.^1^^,^^2^

Early adopters used the concept of “ecological footprint” to challenge prevailing economic assumptions about urban development, which overlooked the full amount of land needed to supply the food, fuel, and other needs of urban populations.^2^ Footprinting soon proliferated as a framework for thinking about usage of fossil fuels, water, and other natural resources.^3^ “‘Footprint’ is now used as a generic term to describe environmental impact,” says Klaus Hubacek, an ecological economist at the University of Maryland. Footprints have been applied to countries, regions, municipalities, corporations, and even individuals.^3^^,^^4^

At the corporate level, “footprints are used to sum up the indirect environmental impacts that occur along a company’s supply chains,” says Tommy Wiedmann, a footprinting expert and associate professor of sustainability research at Australia’s University of New South Wales. They’re useful in a couple of ways, he explains. A footprint analysis may allow a company to influence its suppliers or choose greener suppliers. Footprinting can also show a company where potential financial risks exist. For instance, a high carbon footprint for a company might pose a financial risk if carbon-pricing schemes were to be implemented, Wiedmann says.

Carbon and water footprints, along with other measures of environmental impact, are often incorporated into corporate social responsibility or sustainability reports. Many companies now make this information public in response to increasing demands for transparency from shareholders and consumers.^5^ Some tools currently exist for companies to incorporate chemicals into corporate sustainability analysis as well, but methods of reporting vary from company to company. Until recently there has been no single industry-wide metric—no chemical footprint.^5^

## Tools for Chemicals

It is difficult to define the potential risk posed by a product based on its chemical composition^5^—in terms of both the hazards associated with chemical ingredients and the potential for exposure to those chemicals over the product’s life cycle.^6^

But this information is important, not only because it can foster transparency and discourse around chemicals management, but also because it can create benchmarks against which to measure progress toward safer chemical solutions, says Martin Mulvihill, executive director of the University of California’s Berkeley Center for Green Chemistry. “Lack of transparency and metrics to begin tackling the complex issue of chemicals in the supply chain creates a significant barrier to action for many consumer brands,” he says.

Multiple factors likely contribute to the historical lack of a chemical footprinting framework, says Richard Liroff, an environmental policy expert and founder of the Investor Environmental Health Network, a shareholder advocacy group based in Virginia. A negative reactionary business mentality around chemicals issues—not addressing problematic chemicals until companies are found to be out of compliance—has played a role. In addition, the complexity of weighing a chemical’s diverse environmental health impacts and combining them into one unit is inherently more difficult than accounting for carbon molecules. “Chemicals management has become a much more complicated matter in the past twenty years with the rise in concern over endocrine-disrupting chemicals,” Liroff says.

Liroff coined the term “toxic footprint” in 2009^6^ after advocating for stronger corporate chemicals policies that would shield investors from the type of financial risks posed by government bans or restrictions on products. “We saw that [the concepts of] carbon footprint and water footprint were really gaining currency. We lacked a simple way of communicating with corporate management about chemical issues and how they should be tackled,” he says.

A lack of transparency surrounding chemicals in products and supply chains creates a problem for socially responsible investors.^5^ From an investment perspective, exposure and uncertainty are the main components of risk analysis, says Susan Baker, vice president at Trillium Asset Management, a Boston-based investment management firm focused on socially responsible investing.

Hidden liabilities, which can create uncertainty for the investor, arise when companies either do not know or do not disclose the chemicals in their products and supply chains, Baker says. Ideally, analysts would determine the extent to which chemicals of high concern exist in a company’s supply chains. But Baker says investors have lacked the tools to explore chemical risk in a way that would allow them to compare companies on the basis of chemicals-management performance.

## The Chemical Footprint Project

BizNGO founder Rossi has created a first-of-its-kind tool to assess how companies perform on issues of chemicals management.^7^ The Chemical Footprint Project, formally launched in December 2014, is a collaboration between BizNGO, Clean Production Action, the sustainability consultancy Pure Strategies, and the University of Massachusetts Lowell.^7^

Based loosely on the Carbon Disclosure Project,^8^ an organization that works with businesses and institutional investors to measure and disclose corporate carbon emissions, the Chemical Footprint Project emerged from a set of four principles for safer chemicals that BizNGO created in 2008. Under these principles, companies should 1) know and disclose product chemistry, 2) assess and avoid hazards, 3) commit to continuous improvement, and 4) advocate for public policies and industry standards that support safer chemicals.^9^ “We needed a framework to implement those principles,” Rossi says.

The project defines a company’s chemical footprint as the total mass of chemicals of high concern present in the company’s products and used in its manufacturing operations.^10^ The project uses California’s Candidate Chemicals List to determine chemicals of high concern. California devised the list as part of its Safer Consumer Products regulations, which went into effect in 2013.^11^ The list contains more than 2,300 chemicals that exhibit “a hazard trait and/or an environmental or toxicological endpoint.”^12^

Companies that participate in the Chemical Footprint Project answer a set of 19 questions in four categories and receive a score between zero and 100 based on their answers. The questions explore four areas: 1) how much a company knows about the chemicals of high concern in its products and supply chains, 2) how it implements corporate chemicals policies, 3) what steps a company is taking to reduce those chemicals of high concern, and 4) how much of this information on chemicals the company publicly discloses.^13^

The project itself does not provide tools to screen chemicals of high concern. Instead it evaluates a company’s use of existing tools, such as Clean Production Action’s GreenScreen^®^^14^ or UL’s GreenWERCS™^15^—high-throughput screens that consolidate data on chemical characteristics such as environmental fate and toxicity. These tools allow companies to quickly and efficiently rank the chemicals they use on the basis of their environmental health impacts. “We’re measuring how much of that knowledge around chemicals management that individual companies have,” says Rossi.

Many companies and governments already use such tools to assess existing chemicals, including alternatives to problematic chemicals. The state of Maine, for instance, requires GreenScreen assessment when considering substitutes for toxic chemicals in children’s products.^16^

Eleven companies piloted the Chemical Footprint Project in fall 2014. Ohio-based GOJO Industries—makers of Purell^®^ Advanced Hand Sanitizer—joined the pilot after a business customer approached them about the project. A growing number of GOJO customers, which include major retailers and health care organizations, have adopted environmental purchasing policies that have moved GOJO to think proactively about the chemicals it uses, says Nicole Koharik, global sustainability marketing director for GOJO. “I would say the process of completing the [Chemical Footprint Project] questions and collecting information has led to an increased dialogue about the evolution of product safety internally, among company management, and with external stakeholders,” she says. In 2014 the company published a sustainable chemistry and packaging policy^17^ to guide GOJO and its customers in selecting sustainable materials.

In April 2015 the Chemical Footprint Project made its assessment tools available for free on its website. Participation in the project remains voluntary, as do companies’ decisions to make their scores publicly available.^8^ “We can’t force a company to make its score public, but not making a score public [suggests] hidden liabilities and risk,” says Rossi.

Asset managers currently rank companies on the basis of environmental, social, and governance (ESG) criteria in addition to more traditional indicators of financial performance.^18^ “Investors want to be able to integrate chemical risk into their ESG metrics. The Chemical Footprint Project is the first initiative to create a tool for benchmarking in this fashion,” says Susan Baker of Trillium, who is part of the project’s steering committee.

## Regulation and Risk

The Chemical Footprint Project, Rossi says, has developed against the backdrop of an increasingly complex and splintered governmental regulatory framework for chemicals in the United States and abroad.

Some U.S. states and municipalities have enacted new chemicals safety policies in the absence of reform to the four-decades-old federal Toxic Substances Control Act, which many experts and policy leaders regard as outdated and ineffective.^19^ As of April 2015, 35 states had passed 169 new bills to regulate toxic chemicals.^20^

Although there are direct costs associated with chemicals-related issues such as recalls or product reformulations, damage to brand image may be a bigger financial motivator.^21^ A 2010 analysis estimated that supply chain disruptions linked to sustainability issues (including but not limited to chemicals) cost an average of 0.7% of a company’s total revenue, and that such supply chain disruptions were associated with a 12% decrease in market capitalization, meaning the total value of a publicly traded company’s outstanding shares.^22^ Market capitalization can be used to indicate public perception of a company’s net worth. “Consumer-facing companies are particularly vulnerable to chemical risk due to their high visibility,” Rossi says.

A spate of high-profile cases over the past decade illustrates that vulnerability. A 2007 recall of more than 9 million toys containing lead paint cost toymaker Mattel an estimated $110 million in recall expenses and drove its stock price down 18%. Johnson & Johnson lost nearly 10% of its market share for baby products in China after an advocacy campaign in the United States revealed chemicals of concern—formaldehyde and 1,4-dioxane—in some of its baby products. SIGG USA filed for bankruptcy in 2011 after two years of fighting allegations that they had deceived consumers by failing to disclose the presence of bisphenol A in the lining material of their aluminum water bottles.^21^

“Corporate transparency is a huge issue,” says Rossi, whose background is in environmental policy. How companies engage with public policy surrounding chemicals may be an indicator of underlying practice, he says: “On the one hand, you see companies fighting new regulations, but there are also leading companies supporting regulation and being proactive on chemicals policy.”

## A Mainstream for Green Chemistry?

The Chemical Footprint Project states that its mission is to “transform global chemical use by measuring and disclosing data on business progress to safer chemicals.”^8^ Rossi envisions the initiative creating a “race to the top” in which companies compete against each other to create safer products and greener chemistries.

But simply knowing what chemicals are in products and supply chains doesn’t guarantee that outcome, experts point out, and furthermore, companies could develop “reporting fatigue.” Hubacek says, “There will always be a tradeoff between monetary gains and environmental performance.” But if companies can save money by improving existing chemicals management policies, he adds, they will. In that case, knowing its own chemical footprint can help a company chart where it’s made gains.

A comprehensive footprint analysis allows a company to identify intervention points throughout its supply chain, which gives them leverage to influence suppliers, Hubacek says. He points out, however, that the Chemical Footprint Project does not consider a company’s upstream supply chain. “The ‘system boundary’ is the company, and there is less information about upstream chemicals available,” he says. “Thus, the company in that context has less options to reduce the upstream chemicals.”

Seagate Technology, a maker of hard drives and other electronic data storage devices, already required full disclosure of chemicals of high concern from its suppliers when it participated in the Chemical Footprint Project’s pilot last fall. Such policies have stood Seagate in good stead as the number of regulated substances increased dramatically over the past decade. Every time a new regulation was introduced, Seagate already knew whether the chemicals were in its products, and the company’s data-collection costs have remained relatively flat.^21^^,^^23^

“Materials will always have an impact,” Mulvihill says. “There are no completely benign chemicals.” But an initiative such as the Chemical Footprint Project can help shine light on where to focus research and development efforts, he says. In some cases, removal of high-concern chemicals may be easy—perhaps they aren’t performing an essential function in a product, or there are safer substitutes already available.

Although advances in green chemistry may be a potential benefit of new chemicals management benchmarking frameworks, Mulvihill doesn’t expect innovation to come from existing chemical or material suppliers. “It will be hard, on purely self-interested grounds, for incumbent large players to make shifts toward safer products,” he says.

Such frameworks could, however, provide opportunities for smaller, biobased chemicals manufacturers looking to break into new markets. “An initiative such as the Chemical Footprint Project doesn’t necessarily lead to safer chemicals,” Mulvihill says, “but it’s an important first step in creating a market for those chemicals.”
